# Research on Temperature Compensation Technology for a Flexible Capacitive Pressure Sensing System

**DOI:** 10.3390/mi17060689

**Published:** 2026-06-02

**Authors:** Jianyi Zheng, Shuhan Chen, Zhicheng Xia

**Affiliations:** Pen-Tung Sah Institute of Micro-Nano Science and Technology, Xiamen University, Xiamen 361102, China; chenshuhan@stu.xmu.edu.cn (S.C.); xzcxp99@163.com (Z.X.)

**Keywords:** flexible capacitive pressure sensor array, pressure field sensing, temperature compensation, synchronous detection, time-division multiplexing, aerospace monitoring

## Abstract

Real-time pressure field measurement in aerospace vehicles is challenging because flexible sensor arrays must operate on curved surfaces under coupled thermal and pressure conditions. In this study, a temperature-compensated flexible capacitive pressure sensing system was developed for aerospace applications by integrating an 8 × 8 flexible sensor array, a multi-channel readout circuit based on time-division multiplexing and synchronous detection, and a Particle Swarm Optimization–Backpropagation (PSO-BP) neural network model. Calibration results showed high linearity, with a correlation coefficient of 0.9998 and a maximum relative error of 2.23%. Under coupled temperature–pressure conditions over 5–150 kPa and 10–110 °C, the average measurement error remained below 6%. Flight experiments further demonstrated valid in-flight data acquisition and trend-level pressure variations during key flight events, verifying the feasibility of the proposed approach for distributed aerospace pressure monitoring.

## 1. Introduction

Pressure field measurement in aerospace vehicles is essential for monitoring the operating conditions of critical structures during flight. Components such as external walls, instrument compartments, and interstage sections are subjected to complex aerodynamic loads, transient pressure impacts, and large pressure gradients, which place stringent requirements on sensing systems. Accurate pressure field data are therefore essential for structural evaluation, condition monitoring, and design optimization of aerospace vehicles.

Conventional pressure measurement techniques, including resonant sensors [1,2], pressure-sensitive paint (PSP) [3,4,5], and fiber-optic sensing systems [6,7,8], have been widely investigated for aerospace applications. However, these methods often suffer from limitations in conformal deployment, distributed measurement over curved surfaces, or stable operation under harsh flight environments. In recent years, flexible pressure sensor arrays have shown broad potential in wearable electronics, health monitoring, human–machine interfaces, and flow-related measurements [9,10,11,12]. For aerospace-related sensing, Dong et al. developed an ultrathin all-polyimide flexible skin integrating pressure and airflow sensors for flight parameter estimation [13], while Xiong and co-workers reported conformable capacitive sensor arrays and flexible smart sensing skins for curved-surface aerodynamic perception [14,15,16].

Despite these advances, several challenges remain for aerospace applications. Flexible capacitive sensors are susceptible to temperature-induced drift under coupled thermal and pressure conditions, and practical pressure field monitoring requires a reliable multi-channel readout system for selective addressing and stable measurement of large-scale arrays. These issues highlight the need for integrated sensing, readout, and compensation strategies.

To address these challenges, this study develops a temperature-compensated flexible capacitive pressure sensing system for aerospace vehicles. An 8 × 8 flexible capacitive pressure sensor array and a corresponding multi-channel measurement system based on time-division multiplexing and synchronous detection are designed to enable selective addressing and accurate signal measurement. In addition, a Particle Swarm Optimization–Backpropagation (PSO-BP) neural network model is established to reduce temperature-induced errors by predicting pressure from temperature and capacitance inputs. Calibration experiments, temperature cycling tests, and flight experiments are conducted to evaluate the performance of the proposed system. The results show that the system achieves high linearity and enables effective pressure field monitoring under complex operating conditions.

This work focuses on system-level integration of a flexible capacitive pressure sensor array, a multi-channel readout circuit, and a temperature-compensation model. The main contribution is the demonstration of a complete measurement chain from array sensing and capacitance acquisition to temperature-compensated pressure reconstruction and in-flight validation.

## 2. Materials and Methods

### 2.1. Sensing Principle and Array Design

The sensing mechanism of the flexible capacitive pressure sensor is based on the capacitance variation in a parallel-plate capacitor. The capacitance is determined by the dielectric permittivity, the effective overlap area of the electrodes, and the separation distance between the upper and lower electrodes. When external pressure is applied, the electrode spacing changes, leading to a corresponding variation in capacitance [17,18].

The capacitance C can be expressed as Equation (1):

(1) $$\begin{array}{c} C=\frac{\varepsilon_{0}\varepsilon_{r}A}{d} \end{array}$$

where $\varepsilon_{0}$ is the vacuum permittivity, $\varepsilon_{r}$ is the relative permittivity of the dielectric layer, A is the effective overlap area of the electrodes, and d is the distance between the upper and lower electrodes.

**Figure 1:**
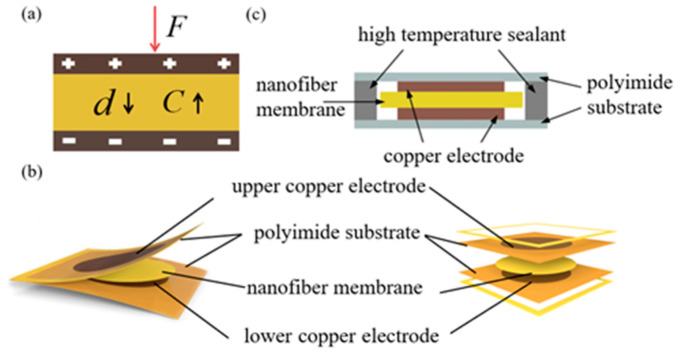
Sensing principle and structure of the flexible capacitive pressure sensor: (**a**) schematic of the parallel-plate capacitive sensing principle, where the arrow indicates the applied pressure direction; (**b**) structure of the flexible capacitive sensing unit, with letters denoting the main structural layers; (**c**) schematic of the vacuum packaging design, where arrows indicate the packaging and sealing direction.

As shown in Figure 1, the sensor consists of polyimide encapsulation layers, copper electrode layers, and a porous nanofiber membrane dielectric layer. To meet the requirements of aerospace applications, a sandwich structure with dual parallel-plate electrodes was adopted. The dielectric layer was a high-temperature-resistant and highly porous electrospun polyimide nanofiber membrane. Electrospun PI nanofiber membranes have been reported as effective porous dielectric layers for flexible capacitive pressure sensors because their loose fibrous structures can improve pressure-induced deformability and sensing performance [19]. The sensor adopted a double-sided parallel-plate sandwich structure, in which the upper and lower copper electrodes were integrated on polyimide encapsulation layers, and shielding electrodes were arranged around the sensing region to reduce external interference. The two polyimide encapsulation layers were bonded using high-temperature adhesive, and the nanofiber membrane was sandwiched between the copper electrodes as the pressure-sensitive dielectric layer. The porous fibrous morphology was selected to improve pressure-induced compressibility, while the polyimide matrix provided thermal stability and compatibility with the encapsulation layers. In this study, the nanofiber membrane was not independently optimized as a standalone material; instead, its functional contribution was evaluated at the packaged-device level through capacitance–pressure calibration, temperature-drift characterization, and in-flight pressure reconstruction.

Pressure-induced changes in the air and dielectric volume fractions may alter the effective dielectric permittivity of the sensing layer, as described by Equation (2), thereby weakening the pressure sensitivity of the sensor.

(2) $$\begin{array}{c} \varepsilon_{eff}=\varepsilon_{air}V_{air}+\varepsilon_{d}V_{d} \end{array}$$

where $\varepsilon_{air}$ is the relative permittivity of air, $V_{air}$ is the volume fraction of air, $\varepsilon_{d}$ is the relative permittivity of the dielectric material, and $V_{d}$ is the volume fraction of the dielectric material.

To suppress air-related permittivity variations and ensure that the capacitance response is mainly governed by pressure-induced structural deformation, the sensor was packaged under vacuum conditions. The polyimide encapsulation further provides good gas barrier performance and thermal stability, which helps maintain sensor reliability under elevated temperatures.

**Figure 2:**
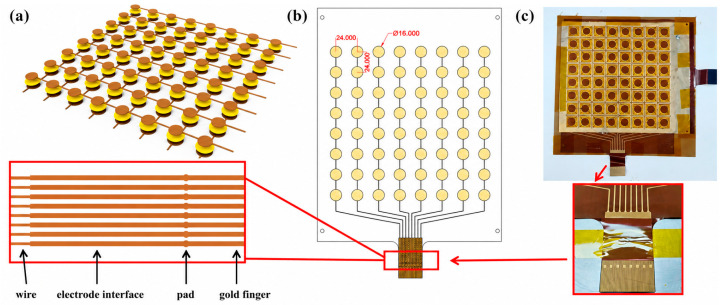
Structure of the 8 × 8 flexible capacitive pressure sensor array: (**a**) array architecture; (**b**) sensing unit geometry; (**c**) photograph of the fabricated array.

As shown in Figure 2, the flexible capacitive pressure sensor adopts an 8 × 8 row–column multiplexed architecture with 64 sensing units. Each sensing unit has a diameter of 16 mm, and the center-to-center pitch between adjacent units is 28 mm. Electrodes in the same row or column share common traces, so that only 16 external connections are required for the entire array.

### 2.2. Temperature Drift Characterization

The temperature-dependent behavior of the flexible capacitive sensor is mainly attributed to three factors. First, temperature variations may change the dielectric permittivity of the nanofiber membrane, leading to capacitance drift. Second, thermal expansion and microstructural instability of the dielectric layer may alter the effective electrode separation and introduce nonlinearity into the capacitance–pressure relationship. Third, the porous dielectric structure is susceptible to moisture adsorption, and the coupled thermal–humidity effect further affects capacitance stability. These factors make temperature drift a significant source of error in pressure measurement.

**Figure 3:**
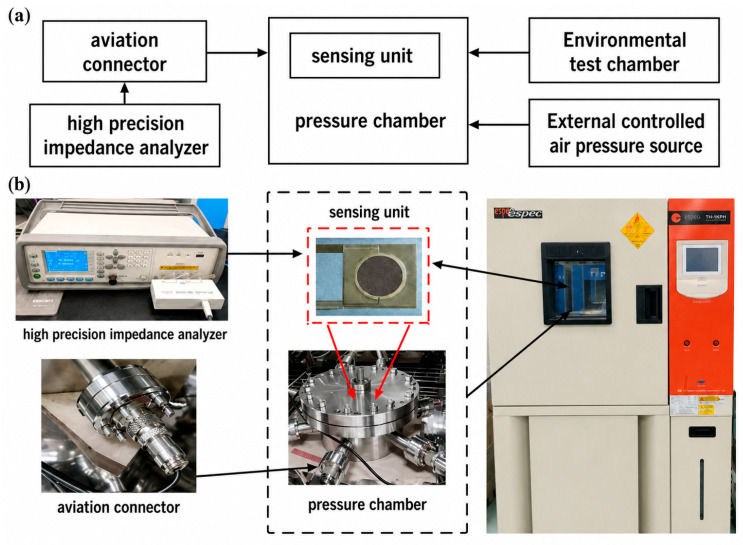
Experimental setup for temperature drift characterization: (**a**) schematic of the test setup; (**b**) detailed view of the experimental environment.

To characterize the temperature drift behavior, the sensing unit was mounted in a hermetic pressure chamber, and its capacitance was monitored in real time using a high-precision impedance analyzer. As shown in Figure 3, the pressure chamber was placed inside an environmental chamber for temperature control. At each target temperature, the system was held for 2 h to ensure thermal equilibrium before measurement. The chamber pressure was then adjusted by an external pneumatic control source, and the capacitance responses of the sensing unit were measured at predefined pressure levels under different temperature conditions. Accordingly, capacitance–pressure curves at different temperatures were obtained.

**Figure 4:**
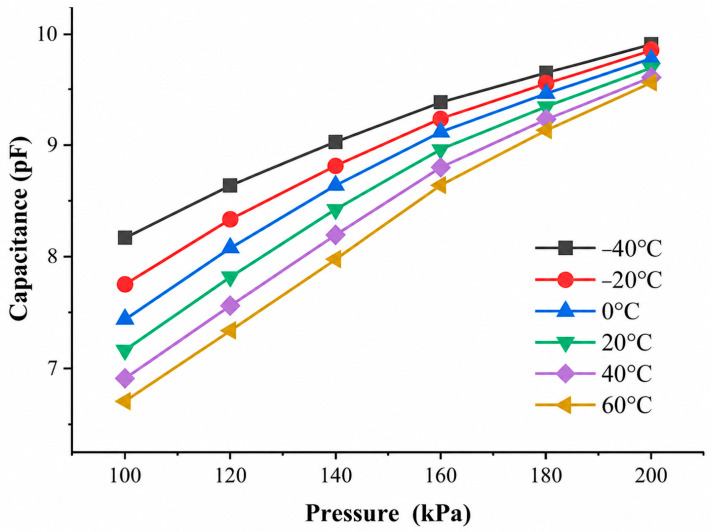
Temperature-induced drift in the capacitance–pressure characteristics of the flexible capacitive sensor.

The measured results are shown in Figure 4. Over the tested temperature range of −40 °C to 60 °C, obvious temperature-dependent drift was observed in the capacitance–pressure characteristics of the flexible capacitive sensor. At a fixed pressure of 100 kPa, increasing the temperature from −40 °C to 60 °C caused a capacitance shift of 2.92 pF, corresponding to more than 20% of the baseline capacitance at 20 °C. By comparison, increasing the pressure from 100 kPa to 200 kPa at room temperature produced a capacitance change of 5.27 pF. These results indicate that temperature-induced drift is substantial and must be considered in practical pressure measurement.

### 2.3. Multi-Channel Measurement System

**Figure 5:**
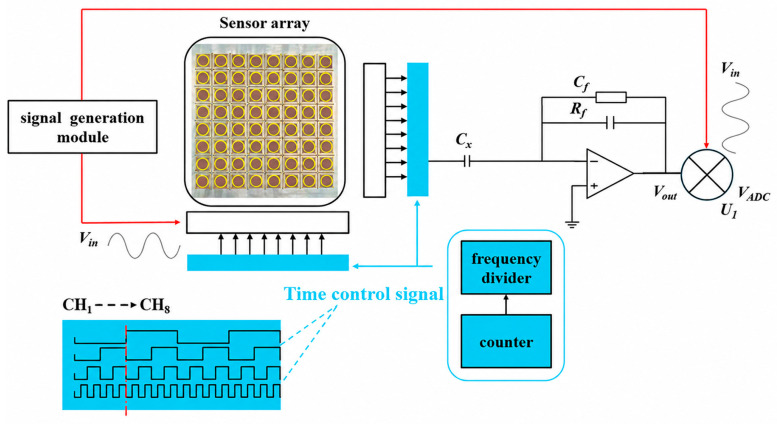
Architecture of the multi-channel measurement system for the flexible capacitive sensor array, where dashed lines indicate time-control signal paths.

As shown in Figure 5, the proposed multi-channel measurement system is designed for the 8 × 8 flexible capacitive pressure sensor array. The system combines a time-division multiplexing network for selective addressing of individual sensing units with a synchronous detection module for accurate capacitance readout.

A sinusoidal excitation signal with an amplitude of 3.5 V and a frequency of 100 kHz is applied to the selected sensing unit, as described by Equation (3). The row and column channels of the array are controlled by a time-division multiplexing network composed of a counter and a frequency-divider circuit. By using sequential control signals, one sensing unit is addressed at a time: the selected row is connected to the excitation source, while the corresponding column is connected to the readout circuit.

(3) $$V_{in}\left(t\right)=A\sin(\omega t)$$

In Figure 5, $C_{x}$ denotes the selected flexible capacitive sensing unit, $C_{f}$ is the feedback capacitor, and $R_{f}$ is the feedback resistor. Two signal paths are defined in the readout circuit: a reference signal and a capacitance-to-voltage converted signal. The converted signal is expressed by Equation (4), where φ represents the phase shift introduced by the capacitance-to-voltage conversion circuit.

(4) $$V_{out}=-\frac{j\omega_{0}R_{f}C_{x}}{1+j\omega_{0}R_{f}C_{f}}A\sin(\omega t+\varphi )$$

The reference signal and the converted signal are then multiplied by the analog multiplier, yielding the multiplier output as given in Equation (5).

(5) $$V_{adc}=\frac{V_{in}\times V_{out}}{U}+Z$$

By combining Equations (4) and (5), the relationship expressed in Equation (6) is obtained.

(6) $$V_{adc}=\frac{C_{x}}{2.5C_{f}}A^{2}\sin(\omega t)\sin(\omega t+\varphi )=\frac{C_{x}}{2.5C_{f}}A^{2}\left(\frac{1}{2}\cos\varphi -\cos(2\omega t+\varphi )\right)$$

In the implemented circuit, $U_{1}$ is set to 2.5 V and $U_{2}$ is set to 0 V. The feedback resistor $R_{f}$ and feedback capacitor $C_{f}$ are chosen as 1 MΩ and 30 pF, respectively. Experimental characterization shows that the phase deviation introduced by the capacitance-to-voltage conversion circuit is approximately 177°. After low-pass filtering, the second-harmonic component is attenuated and the final processed output is obtained, as described by Equation (7).

(7) $$V_{adc}^{\prime }=\frac{C_{x}}{2.5C_{f}}A^{2}\cos(177{}^{\circ})$$

Under fixed excitation amplitude, excitation frequency, and circuit parameters, the final output signal is determined by the capacitance of the selected sensing unit. The synchronous detection module uses the AD734 four-quadrant analog multiplier (Analog Devices, Wilmington, MA, USA) and the OPA828 operational amplifier (Texas Instruments, Dallas, TX, USA) to enable stable readout of the flexible capacitive sensor array. It should be noted that the 100 kHz signal is the excitation frequency used for capacitance readout, whereas the effective in-flight data transmission rate is determined by the onboard data-acquisition and telemetry chain.

### 2.4. PSO-BP Model and Experimental Procedure

To reduce the influence of temperature-induced drift on pressure measurement, a Particle Swarm Optimization–Backpropagation (PSO-BP) neural network model was introduced. Polynomial fitting is often insufficient for describing the nonlinear relationship among temperature, capacitance, and pressure. Therefore, a PSO-BP neural network was introduced to optimize the initial weights and thresholds of the BP model and improve pressure prediction under coupled temperature–pressure conditions [20,21].

**Figure 6:**
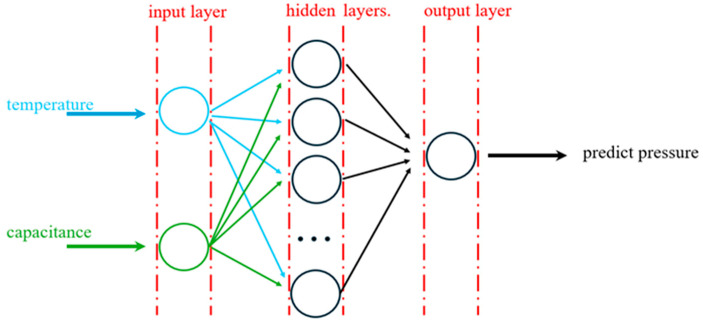
Structure of the PSO-BP neural network used for pressure prediction, where circles represent neurons and arrows indicate information flow.

As shown in Figure 6, the PSO-BP model adopts a multilayer feedforward neural network structure. In this study, temperature and capacitance were used as the two input variables, and pressure was used as the single output variable. During training, the initial weights and thresholds of the BP neural network were optimized using the PSO algorithm to improve convergence and reduce the risk of local minima.

A total of 2562 sets of pressure measurement data were obtained under different temperature conditions. The dataset was divided into training, testing, and validation subsets at a ratio of 9:0.5:0.5. The PSO algorithm was used to optimize the initial weights and thresholds of the BP network before backpropagation training. In the present study, the swarm size was set to 20, and the two learning factors were both set to 2.0. The final network architecture is listed in Table 1. The hidden-layer sizes were determined empirically through repeated training trials to balance prediction accuracy and overfitting risk.

**Figure 7:**
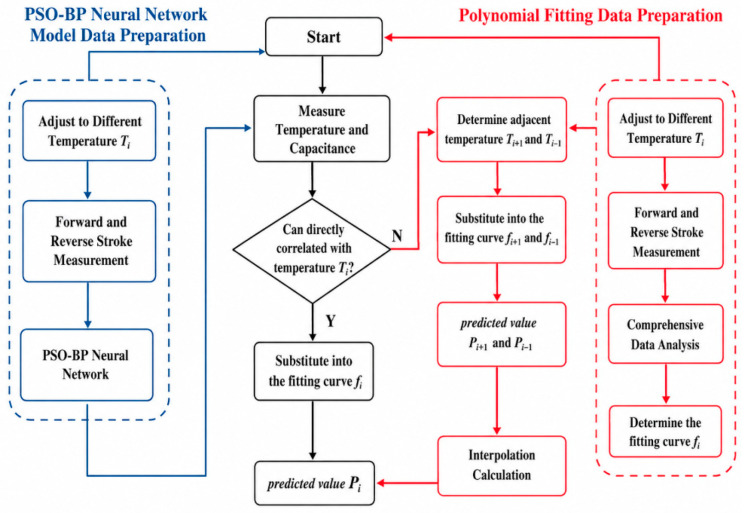
Comparison of data-processing procedures using polynomial fitting and the PSO-BP model.

To evaluate model performance, the coefficient of determination ($R^{2}$) and the root mean squared error (RMSE) were used as fitness metrics. The $R^{2}$ value is calculated by Equation (8), and the RMSE is calculated by Equation (9).

(8) $$R^{2}=1-\frac{\sum_{i=1}^{n}{\left(y_{i}-{\hat{y}}_{i}\right)}^{2}}{\sum_{i=1}^{n}{\left(y_{i}-\bar{y_{i}}\right)}^{2}}$$

(9) $$RMSE=\sqrt{\frac{1}{n}\sum_{i=1}^{n}{\left(y_{i}-{\hat{y}}_{i}\right)}^{2}}$$

where $y_{i}$ is the reference value, ${\hat{y}}_{i}$ is the predicted value, $\bar{y}$ is the mean of the reference values, and n is the number of samples. After model training, the trained PSO-BP network was used to predict pressure from measured temperature and capacitance data. The predicted results were then compared with reference pressure values obtained from the calibration experiments. The overall data-processing framework of the PSO-BP approach is illustrated in Figure 7.

## 3. Results

### 3.1. Calibration of the Capacitance Readout Circuit

**Figure 8:**
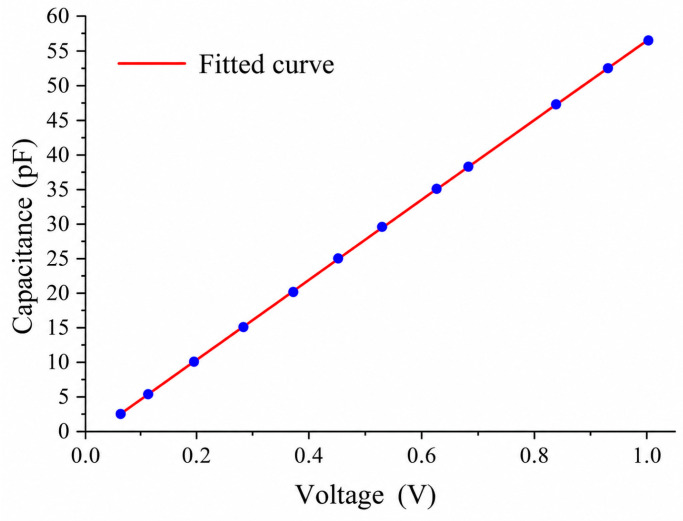
Voltage–capacitance calibration curve of the home-made capacitance readout circuit, where blue dots represent measured data and the solid line represents the linear fit.

To evaluate the capacitance measurement accuracy of the home-made capacitance readout circuit, rather than the complete pressure sensing system, a capacitor array composed of precision surface-mount capacitors was used as the device under test. The reference capacitances were measured using a high-precision LCR meter under the same excitation conditions as the proposed readout circuit. The output voltages of the proposed readout circuit were then recorded. A linear fitting was performed between the reference capacitance values and the output voltages, yielding the calibration relationship shown in Figure 8 and Equation (10).

(10) $$C=57.48144\times V_{ad}-1.17922$$

The fitting result exhibited high linearity, with a correlation coefficient of 0.9998. Based on the calibration curve, the fitted capacitance values were calculated from the measured output voltages, and the relative errors were determined by comparison with the LCR-measured reference capacitance values, as defined by Equation (11).

(11) $$RE=\left|\frac{C_{Fit}-C_{Ref}}{C_{Ref}}\right|\times 100\%$$

where $C_{Fit}$ is the fitted capacitance calculated from the output voltage of the readout circuit, and $C_{Ref}$ is the reference capacitance measured by the commercial LCR meter.

The detailed calibration results are listed in Table 2. Over the tested capacitance range, the maximum relative error between the fitted capacitance and the LCR-measured reference capacitance was 2.23%, indicating that the home-made capacitance readout circuit achieved good agreement with the commercial LCR meter.

### 3.2. Temperature Compensation Performance of the PSO-BP Model

**Figure 9:**
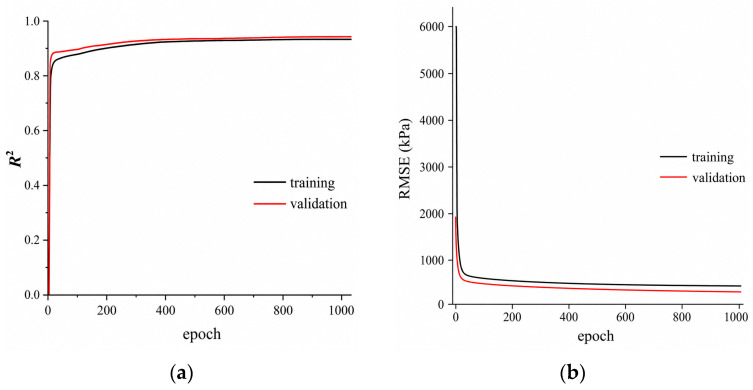
Training performance of the PSO-BP model: (**a**) coefficient of determination $R^{2}$; (**b**) root mean square error (RMSE).

The training and testing performance of the PSO-BP model is shown in Figure 9. As the number of training iterations increased, the coefficients of determination for both the training and testing sets gradually converged, indicating improved fitting performance. After 1053 training iterations, the coefficient of determination (R^2^) reached 0.9421. Meanwhile, the RMSE values of the training and testing sets gradually decreased and stabilized, indicating steady convergence of the model. It should be noted that these metrics were used to evaluate the fitting performance of the PSO-BP model during training, whereas the average measurement error reported later reflects the pressure reconstruction accuracy of the overall sensing system under experimental conditions. Therefore, the two types of metrics describe different aspects of performance.

**Figure 10:**
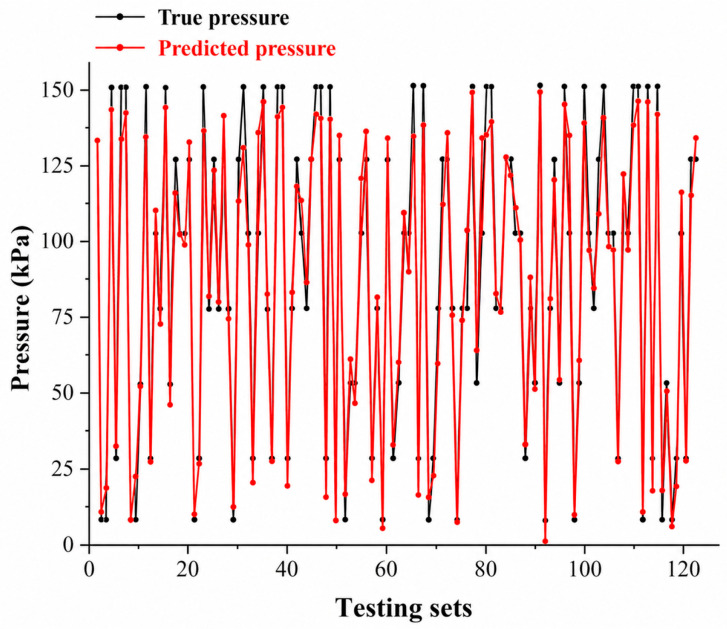
Comparison between predicted and reference pressure values in the testing set.

The prediction performance of the trained PSO-BP model on the testing set is shown in Figure 10. The predicted pressure values followed the reference trend over the full measurement range, indicating that the model captured the nonlinear temperature–capacitance–pressure relationship. Larger deviations near 5 kPa and 150 kPa suggest that future work should further optimize boundary-condition sampling.

Compared with conventional polynomial fitting, the PSO-BP model more effectively captured the nonlinear coupling among temperature, capacitance, and pressure, particularly near the boundaries of the measurement range.

### 3.3. In-Flight Pressure Measurement Results

The proposed pressure measurement system was validated in a launch-vehicle flight experiment. A total of 175 s of valid in-flight data were obtained. The flight-test system consisted of the launch vehicle, payload platform, and ground receiving system. The proposed pressure-field measurement system was integrated into the payload platform and transmitted data to the ground station through the telemetry channel. The payload platform was installed in the interstage section of the launch vehicle, approximately 300 mm above the lower end of the interstage section. The flexible capacitive sensor array was mounted on the inner wall of the payload platform, and ventilation openings were arranged on the side wall of the platform to allow gas exchange between the platform interior and the surrounding compartment environment. The readout circuit and data-acquisition module were fixed inside the payload platform and connected to the sensor array through the system interface and signal harness. The onboard electronics included the multi-channel capacitance readout circuit, an RS485 communication interface, and a data-acquisition module. Each data packet contained the sensing-unit information and the corresponding digitized output signal, allowing reconstructed pressure values to be mapped back to the spatial positions of the 8 × 8 array.

Before reconstructing the in-flight pressure field, three abnormal sensing units were excluded according to the pre-flight calibration and payload–environment calibration results, and the remaining 61 valid units were used for subsequent analysis. These abnormal units exhibited basic errors greater than 10%, and their positions were consistent in the high-temperature, low-temperature, and payload-environment calibration tests. Therefore, they were identified before flight-data reconstruction rather than treated as random in-flight failures.

A preliminary root-cause analysis suggests that the abnormal responses were mainly associated with local fabrication and packaging inconsistencies. The porous nanofiber dielectric membrane is highly sensitive to local deformation; therefore, micro-wrinkles generated during manual fabrication may lead to nonuniform dielectric stiffness after encapsulation. In addition, uneven distribution of the high-temperature adhesive and local degradation of the vacuum package may further affect the effective electrode spacing and pressure-induced capacitance variation. A vibration reliability test was also conducted before the flight test, in which the measurement system remained powered and the output signals were monitored under stepped vibration loading. The measured capacitance variation remained within 1%FS below 15 g and within 3%FS even at the limiting vibration level of 17 g. These results suggest that the abnormal units were more likely related to fabrication and packaging consistency than to random vibration-induced electrode delamination or shear damage during flight.

**Figure 11:**
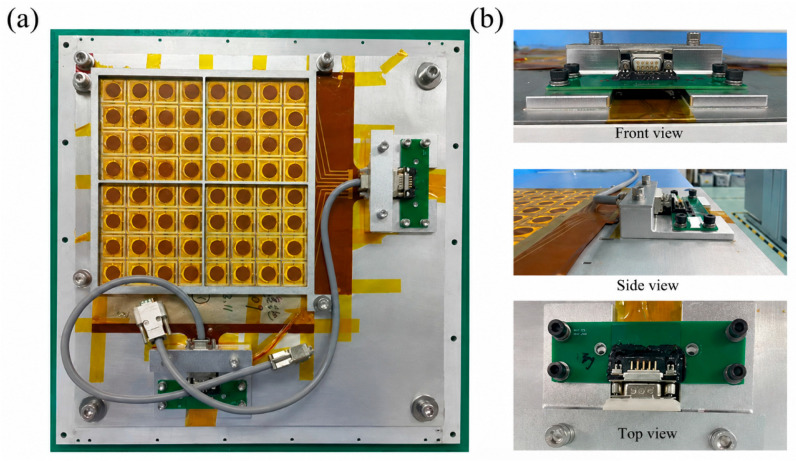
Assembly and connector interface of the flexible capacitive pressure sensing array: (**a**) assembled sensor array; (**b**) front, side, and top views of the connector interface.

Figure 11 shows the assembly and connector interface of the flexible capacitive pressure sensing array. The images illustrate the fabricated 8 × 8 sensor array, its assembled configuration, and the front, side, and top views of the mechanical connector interface used for signal transmission and system integration.

**Figure 12:**
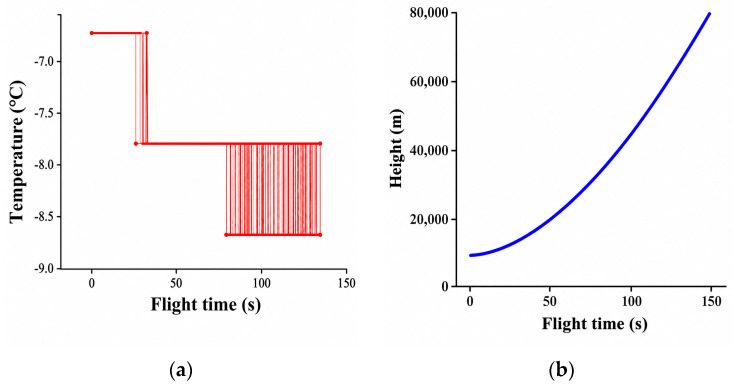
In-flight environmental data: (**a**) internal temperature; (**b**) flight altitude.

The in-flight environmental data are shown in Figure 12. During the effective flight period, the internal temperature of the payload platform remained within a relatively narrow range, whereas the flight altitude increased continuously until stage separation. These environmental data provide the flight-condition background for interpreting the reconstructed pressure response.

During the flight test, the internal environmental temperature of the payload platform was measured by a commercial aerospace-grade temperature sensor installed near the pressure-field measurement system. Before flight-data reconstruction, the temperature channel was checked together with the payload telemetry system to ensure valid data transmission. The measured temperature was used as the temperature input of the PSO-BP compensation model for in-flight pressure reconstruction. The flexible sensor array and the temperature sensor were both located inside the payload platform; therefore, the measured temperature was used to represent the local thermal environment of the valid sensing units during the effective flight period. Since the recorded internal temperature varied only within a relatively narrow range, this single-point temperature input was considered acceptable for the present flight-data analysis. Nevertheless, the current configuration provides limited spatial temperature resolution. Future versions of the system will integrate distributed temperature sensors near the pressure array to improve local temperature compensation under nonuniform or rapidly varying thermal fields.

**Figure 13:**
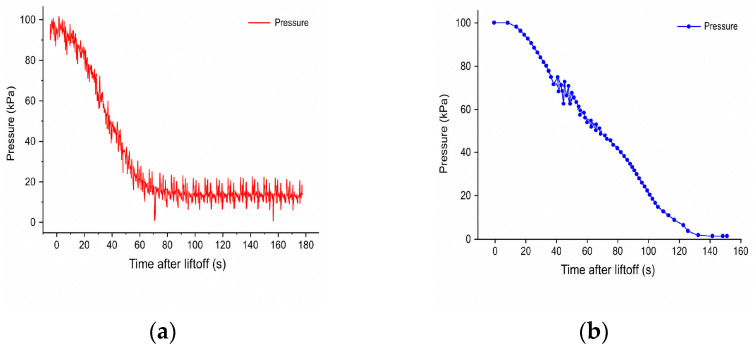
Comparison of in-flight pressure trends: (**a**) pressure trend reconstructed by the proposed flexible capacitive pressure sensing system; (**b**) pressure trend measured by the commercial high-temperature pressure sensor installed on the same launch-vehicle platform.

Based on the temperature-compensated pressure reconstruction, the overall trend of the in-flight pressure data measured by the 61 valid sensing units is shown in Figure 13a. Before liftoff, the measured pressure was close to atmospheric pressure, and it decreased progressively after launch. For comparison, Figure 13b shows the pressure trend measured by a commercial high-temperature pressure sensor installed on the same launch-vehicle platform. The commercial sensor was mounted outside the compartment and acquired pressure data at 10 Hz, whereas the proposed flexible sensing system was installed inside the payload platform and had an effective in-flight data rate of approximately 1 Hz due to telemetry bandwidth limitation. Therefore, the comparison was used to evaluate trend consistency rather than point-by-point numerical agreement. Both systems captured the initial atmospheric pressure level and the decreasing pressure trend during ascent, supporting the feasibility of the proposed system for in-flight pressure-field monitoring.

To further clarify the comparison with the commercial reference sensor, the main differences between the two systems are summarized in Table 3.

**Figure 14:**
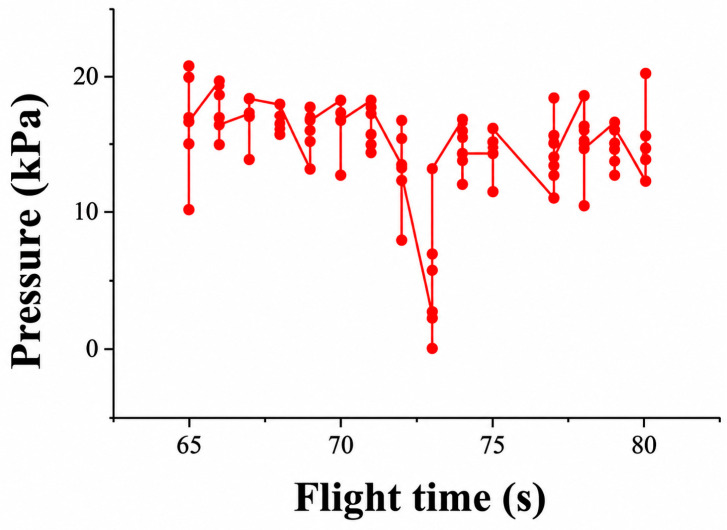
Zoomed-in view of the in-flight pressure data around the stage-separation-related event, where red dots represent measured data points and red lines indicate pressure variation.

A local segment of the in-flight pressure data is shown in Figure 14. Around 71–73 s after liftoff, multiple sensing units exhibited a step-like pressure drop from approximately 14 kPa to 2 kPa, coinciding with the stage-separation-related event. Owing to the approximately 1 Hz telemetry-limited data rate, the instantaneous transition could not be fully resolved, but the measured data still captured the main trend-level pressure response.

Representative sensing units exhibited consistent pressure evolution during flight, further supporting the stability of the array-based measurement.

**Figure 15:**
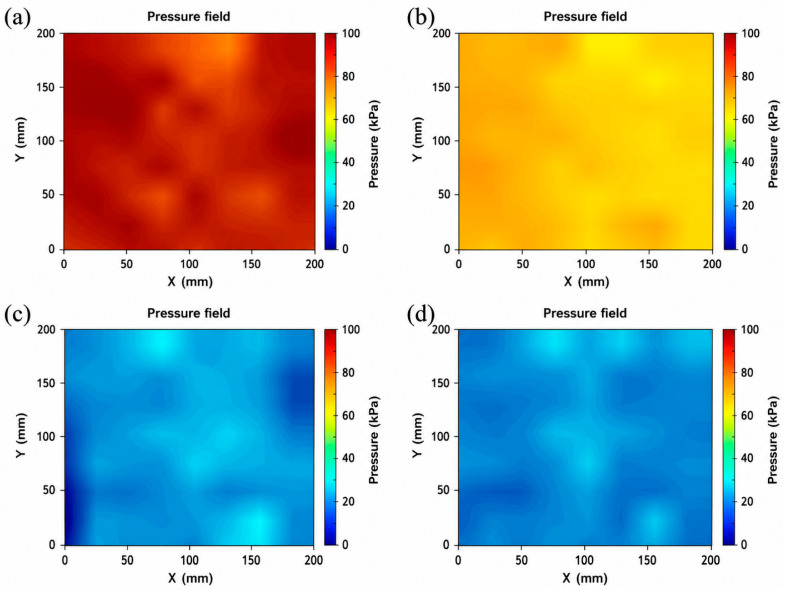
Spatial pressure-field maps reconstructed from the 8 × 8 flexible capacitive sensor array during flight: (**a**) 0–5 s; (**b**) 25–30 s; (**c**) 70–75 s; (**d**) 140–145 s.

To further demonstrate the spatially resolved capability of the proposed array, the in-flight pressure data were reconstructed into pressure-field maps, as shown in Figure 15. The spatial coordinates of the 8 × 8 sensing units were used to map the pressure values onto the array plane. The three abnormal units identified from the calibration results were masked, and the remaining 61 valid units were used for pressure-field reconstruction. Representative flight periods, including 0–5 s, 25–30 s, 70–75 s, and 140–145 s, were selected to illustrate the evolution of the distributed pressure field. The reconstructed maps show that the overall pressure level decreased during flight, which is consistent with the time-series pressure trend. These results demonstrate that the proposed system can provide distributed pressure-field information rather than only single-point or averaged pressure values.

## 4. Discussion

The results demonstrate that the proposed temperature-compensated flexible capacitive pressure sensing system enables pressure-field monitoring under laboratory and in-flight conditions. Unlike studies focusing mainly on sensing materials, flexible skins, or benchtop characterization, this work emphasizes system-level integration of an 8 × 8 flexible sensor array, a multi-channel readout circuit, and a PSO-BP compensation model. The system achieved a capacitance calibration correlation coefficient of 0.9998, a maximum relative error of 2.23%, and an average pressure reconstruction error below 6% under coupled temperature–pressure conditions. These results indicate that the proposed measurement chain can support temperature-compensated distributed pressure monitoring in aerospace environments.

The PSO-BP model improved pressure prediction by capturing the nonlinear coupling among temperature, capacitance, and pressure. Compared with polynomial fitting and previously reported data-driven compensation methods, it provides a direct mapping from measured temperature and capacitance to reconstructed pressure, simplifying compensation for the flexible sensor array.

The current PSO-BP architecture prioritizes pressure-reconstruction accuracy for offline processing, but its size is relatively large for onboard edge computing. Lightweight architectures, fewer hidden neurons, model pruning, or knowledge distillation may reduce computational cost with limited accuracy loss. Future work should balance compensation accuracy and embedded implementation efficiency.

Mechanical hysteresis is another important factor affecting flexible capacitive pressure sensors under cyclic loading. In the present calibration procedure, loading and unloading data were used to evaluate the basic error of the sensing units, although a dedicated recovery-time test was not conducted. The larger loading–unloading difference under high- and low-temperature conditions suggests viscoelastic deformation and delayed recovery of the porous nanofiber membrane and polymer encapsulation. Future work should quantify hysteresis error and recovery time under cyclic and dynamic aerospace conditions.

The in-flight experiment further demonstrates the applicability of the proposed system. A total of 175 s of valid pressure data were obtained, and pressure variations associated with key flight events were successfully captured. Although the telemetry bandwidth limited the temporal resolution, the measured pressure evolution remained consistent with the overall flight process and showed similar trend characteristics to those obtained from a commercial high-temperature pressure sensor installed on the same platform. Therefore, the comparison is intended to verify consistency in trend and event response rather than strict numerical equivalence.

Nevertheless, several limitations remain. Although the capacitance readout circuit uses a 100 kHz excitation signal for capacitive sensing, the effective in-flight data rate of the present flight experiment was limited by the telemetry bandwidth and data-packet transmission configuration. Therefore, the approximately 1 Hz data rate reflects the limitation of the flight telemetry chain rather than the intrinsic excitation or readout capability of the capacitive circuit. This flight experiment was mainly intended to verify the feasibility of temperature-compensated distributed pressure-field monitoring under a real launch-vehicle environment, rather than to fully resolve high-frequency aerodynamic pressure fluctuations. As a result, rapid pressure transitions, such as those occurring during stage-separation-related events, could only be captured as trend-level changes rather than fully time-resolved transient waveforms. Future implementations should introduce onboard buffering, higher-rate local storage, edge preprocessing, event-triggered data compression, or Integrated Network-Enhanced Telemetry (iNET)-based transmission to improve the reconstruction of high-dynamic pressure events.

The temperature-drift characterization was performed under quasi-static thermal equilibrium conditions to establish a reliable capacitance–pressure–temperature mapping for compensation-model training. This protocol was appropriate for the present flight-data reconstruction because the recorded internal temperature of the payload platform varied only within a narrow range during the valid flight period. However, rapid thermal transients in aerospace environments may cause thermal lag among the temperature sensor, sensing unit, encapsulation layer, and surrounding structure, leading to additional compensation errors. Therefore, the present results should be interpreted as validation under a relatively stable internal thermal environment rather than as full verification under severe thermal-shock conditions. Future work will include dynamic temperature-ramping experiments, distributed temperature sensing near the pressure array, and transient thermal-lag modeling.

In addition, several sensing units exhibited abnormal responses during flight, suggesting that further improvements in fabrication consistency, electrical interconnection reliability, and packaging robustness are still needed. Because the present work focuses on system-level pressure-field measurement and temperature compensation, the porous polyimide nanofiber membrane was evaluated primarily within the packaged capacitive sensing unit rather than as a standalone material. The calibration and flight-test results indicate that the membrane provided sufficient pressure-induced deformability and thermal compatibility for the proposed sensing system. However, further membrane-level characterization, including morphology, porosity, dielectric properties, compressive recovery, and thermomechanical stability, would be valuable for quantitatively correlating the dielectric microstructure with sensitivity, hysteresis, and long-term reliability.

## 5. Conclusions

In this study, a temperature-compensated flexible capacitive pressure sensing system for aerospace vehicles was developed by integrating an 8 × 8 flexible sensor array, a multi-channel readout circuit, and a PSO-BP neural network model. The proposed system showed high calibration linearity, with a correlation coefficient of 0.9998 and a maximum relative error of 2.23%. Under coupled temperature–pressure conditions, the average measurement error remained below 6%, indicating that the proposed compensation strategy can effectively suppress temperature-induced drift. In addition, 175 s of valid in-flight data were obtained, and pressure variations associated with key flight events were successfully captured. These results demonstrate the feasibility of the proposed system for distributed aerospace pressure monitoring.

## Figures and Tables

**Table 1:** Architecture of the PSO-BP neural network for pressure prediction.

Layer	Number of Neurons
Input Layer	2
Hidden Layer 1	99
Hidden Layer 2	198
Hidden Layer 3	99
Output Layer	1

**Table 2:** Quantitative comparison between the home-made capacitance readout circuit and the commercial LCR meter.

Nominal Capacitance (pF)	LCR-Measured Capacitance (pF)	Readout Voltage (V)	Fitted Capacitance (pF)	Relative Error (%)
2	2.46	0.0631	2.45	0.49%
5	5.29	0.1146	5.41	2.23%
10	10.04	0.1952	10.04	0.01%
15	14.97	0.2839	15.14	1.13%
20	20.07	0.3734	20.28	1.07%
25	25.09	0.4528	24.85	0.96%
30	29.53	0.5296	29.26	0.91%
35	35.02	0.6268	34.85	0.49%
38	38.00	0.6812	37.98	0.06%
47	47.12	0.8373	46.95	0.36%
52	52.24	0.9310	52.34	0.18%
56	56.11	1.0016	56.39	0.51%

**Table 3:** Comparison between the proposed system and the commercial high-temperature pressure sensor.

Item	Proposed System	Commercial Sensor
Sensing mode	8 × 8 flexible capacitive array	Single-point pressure sensor
Installation	Inside the payload platform	Outside the compartment
Data rate	Approximately 1 Hz, telemetry-limited	10 Hz
Function	Pressure-field reconstruction	Reference trend comparison

## Data Availability

The data presented in this study are not publicly available due to project confidentiality restrictions but may be available from the corresponding author upon reasonable request.

## Abbreviations

The following abbreviations are used in this manuscript:

PSO	Particle Swarm Optimization
BP	Backpropagation
PSO-BP	Particle Swarm Optimization–Backpropagation
RMSE	Root Mean Square Error
$R^{2}$	Coefficient of Determination
LCR	Inductance–Capacitance–Resistance
